# Association of *ApaI *and *TaqI* polymorphisms in *VDR* Gene with Breast Cancer

**DOI:** 10.31557/APJCP.2020.21.9.2667

**Published:** 2020-09

**Authors:** Amir Hassan Matini, Negar Jafarian-Dehkordi, Banafshe Bahmani, Mehran Sharifi, Danial Jahantigh, Tahereh Mazoochi

**Affiliations:** 1 *Department of Pathology, School of Medicine, Kashan University of Medical Sciences, Kashan, Iran. *; 2 *Department of Hematology and Oncology, School of Medicine, Isfahan University of Medical Sciences, Isfahan, Iran. *; 3 *Department of Biology, Faculty of Science, University of Sistan and Baluchestan, Zahedan, Iran. *

**Keywords:** Breast cancer, vitamin D receptor, genetic polymorphism, ApaI, TaqI

## Abstract

**Background:**

Vitamin D inhibits cell proliferation via the vitamin D receptor (VDR), which may affect breast cancer risk. This study aimed to investigate the association of ApaI and TaqI polymorphisms of the *VDR* gene with breast cancer risk which followed by stratified analysis.

**Materials and methods:**

A case-control study was conducted on 150 breast cancer patients and 150 healthy controls. *VDR ApaI *and* TaqI* genotyping were performed by PCR-RFLP. Some demographic and pathologic features of patients were extracted from their archived files and then were analyzed by genotypes distributions.

**Results:**

For ApaI polymorphism, our data showed a significant difference between the patient and healthy groups for mutant allele carriers compared with those with AA genotype. Besides, statistical analysis showed that there was a significant association between the C allele and the increased risk of breast cancer. For TaqI polymorphism, statistical analysis revealed that there was a significant association between CC genotype and increased risk of breast cancer. Also, there was a significant association between the C allele and the increased risk of breast cancer. In a preliminary study, stratified analysis based on the size of tumor and lymph node metastasis revealed no significant association between two* ApaI* and *TaqI* variations and these parameters.

**Conclusions:**

Based on our results, the *VDR* *ApaI *and* TaqI* variations could be considered as genetic risk factors for breast cancer. However, further studies with a larger sample size are required to obtain more accurate outcomes, especially in stratified analysis.

## Introduction

Breast cancer is the major cause of death in women worldwide. It is responsible for 16% of all and 22% of invasive female cancers. This cancer is diagnosed as the most frequent cancer among Iranian women (Parkin et al., 2003; Taghavi et al., 2012). Variations in some genes which involved in growth, differentiation, and apoptosis, may influence breast cancer susceptibility (Vogelstein and Kinzler, 2004). Although a specific gene responsible for the familial breast cancer is not discovered yet mutations in some suppressor genes such as *BRCA1, BRCA2, ATM, PTEN, TP53, BRIP1, PALB2, NBS1, RAD50, MSH2, MLH*, and *CHEK2 *are occurred in about half of the familial breast cancer (Walsh and King, 2007). 

Apoptotic and anti-proliferative impacts of vitamin D against different malignancies such as breast cancer have been previously studied (Crew, 2013). Many studies investigated the effects of vitamin D on breast cancer and confirmed the protective role of vitamin D in this disease (Stoica et al., 1999; Jensen et al., 2001; Crew, 2013). The effects of vitamin D, 1α, 25-dihydroxyvitamin D3 (1,25(OH)2D3), are through the vitamin D receptor (VDR) which expressed in most cell types, enclosing breast tissues (Townsend et al., 2005). The active form of vitamin D regulates growth, differentiation, and apoptosis that is mediated by the vitamin D receptor (VDR) (Evans, 1988). VDR is a member of the nuclear receptor family of steroid hormones that acts as a transcriptional regulatory factor in most tissues (Jurutka et al., 2001). Also, it is reported that vitamin D receptors can play an important role in the pathogenesis of breast cancer (Shen and Brown, 2003; Swami et al., 2003). Genetic variants in the *VDR *gene may decrease the levels of VDR expression and increase the risk of breast tumors (Lopes et al., 2010). 

*VDR* gene is located in chromosome 12 (12q13.11) which comprised 11 exons with some common single nucleotide polymorphisms such as* ApaI* and* TaqI*. The *ApaI *variable site (rs7975232; g.48238837C>A) is located in intron 8 of the *VDR *gene and the TaqI (rs731236; g.48238757A>G) variation is also located in the same intron. We focused on the distribution of VDR ApaI and TaqI single nucleotide polymorphisms (SNPs) in Isfahan province breast cancer patients compared to a healthy population. 

This study aimed to investigate the association of the mentioned polymorphisms with breast cancer risk in combination with demographic and pathologic features.

## Materials and Methods

*Subjects and blood samples collection*

A case-control study was conducted to investigate the association of ApaI and TaqI polymorphisms in the VDR gene with breast cancer risk. The 300 samples consisting of 150 patients with breast cancer as a case group and 150 healthy women as a control group were included in this study. All subjects were selected from women who referred to Hospitals in Isfahan province (Isfahan, Iran). Women with breast cancer were assessed based on the clinical examinations as well as pathological and mammographic examinations. Also, the breast cancer patients had not been exposed to chemotherapy and/or radiotherapy. The women included in the control group were healthy individuals referring to the same hospital for routine tests and they had no history of breast cancer. Finally, blood samples were collected into sterile tubes containing anticoagulant EDTA sodium salt. Written informed consent obtained from all subjects. The study approved by the Medical Ethics Committee of Kashan University of Medical Sciences.

*DNA extraction, PCR-RFLP, and DNA sequencing*

The genomic DNA extracted from the collected blood samples by a commercial kit according to the manufacturer’s procedure. Genotypes of VDR-ApaI and -TaqI were detected by polymerase chain reaction-restriction fragment length polymorphism (PCR-RFLP) method. The sense and antisense primers for amplifying the fragment containing both ApaI and TaqI polymorphisms were designed by Oligo7 software (DBA Oligo, Inc., USA). The PCR combination was consist of the following reagents: 10μl of 2X Taq PCR Premix, 0.2 µM each of forward and reverse primers, and 30 ng of extracted DNA up to a total volume of 20 µl. All PCR reagents were purchased from CinnaGen Company (CinnaGen, Tehran, Iran). PCR was done in a Peqlab thermal cycler (peqSTAR, Germany) with a program that was shown in table 1. To detection of VDR-ApaI and -TaqI variations, the PCR products were respectively treated by ApaI (Fermentas GmbH, Leon-Rot, Germany) and TaqI (Fermentas GmbH, Leon-Rot, Germany) restriction endonucleases based on the manufacturer’s protocol. To confirm the PCR-RFLP results, 5% of PCR products with different genotypes were sequenced by Bioneer Company (Bioneer, South Korea). The sequence data were analyzed by the Chromas software ver. 2.4.4.

**Figure 1:**
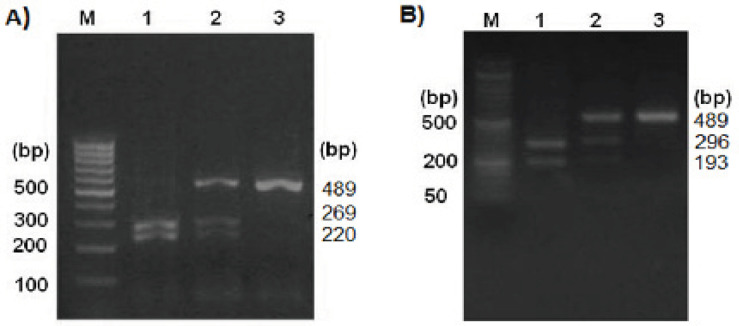
Detection of Genotypes of ApaI and TaqI Polymorphisms by PCR-RFLP. A) The pattern of agarose gel indicated genotypes CC (lane 1), AC (lane 2), and AA (lane 3). B) The pattern of agarose gel indicated genotypes CC (lane 1), TC (lane 2), and TT (lane 3)

After the PCR procedure, a fragment with 489-bp length containing two ApaI and TaqI SNPs was amplified. This fragment was treated individually once with ApaI and once with TaqI restriction enzymes. Regarding the ApaI polymorphism, the results showed that the 489-bp fragment in some samples was digested into two 220- and 269-bp fragments, and in some samples, it was digested into three 489-, 269- and 220-bp fragments. In some samples, however, only one band containing the 489-bp fragment was observed because there was no restriction site for ApaI in these fragments. Samples showing only one band on agarose gel have the AA genotype. Samples with two bands had the CC genotype and samples with three bands had the AC genotype (Figure 1). About the TaqI polymorphism, the results indicated that the 489-bp fragment in some samples was digested into two 296- and 193-bp fragments, and in some samples, it was digested into three 489-, 296- and 193-bp fragments. In some cases, however, only one band containing the 489 bp fragment was observed because there was no restriction site for TaqI in these samples. Samples showing only one band on agarose gel have TT genotype. Samples with two bands had CC genotype and samples with three bands had TC genotype (Figure 1). Data from DNA direct sequencing indicated that the fragment sequence was bona fide at ApaI and TaqI positions.

*Statistical analysis*

All of the statistical analyses were done by SPSS version 20 (SSPS Inc., USA). Quantitative variables were analyzed by an independent t-test. Hardy-Weinberg equilibrium (HWE) was checked by the chi-square test for both case and control groups. Differences in allele and genotype frequencies distribution and between control and case groups were evaluated by utilizing a chi-square test. Odds ratio (OR) and 95% confidence interval (CI) were estimated to evaluate the association of different alleles and genotypes with breast cancer and its subgroups (Talebi et al., 2018). 

## Results

*Association of VDR-ApaI and -TaqI polymorphisms with breast cancer risk*

This study included a total of 150 healthy women and 150 age-matched breast cancer subjects, that there were no significant differences in body mass index (BMI), and menopause status between two studied groups. Our data showed that the distribution of VDR genotypes for both SNPs was met Hardy-Weinberg equilibrium (HWE) in both the case and control groups.

For ApaI polymorphism, alleles with adenine nucleotide were more abundant than other allele. The frequency distribution of AC genotype in the healthy and diseased groups was 40.00% and 48.67%, respectively, while this ratio for CC mutant genotype was 12.00% and 16.00%, respectively. Statistical analysis showed that there was a significant association between AC genotype and increased risk of breast cancer (Table 2). Statistical analysis showed a significant difference between the patient and healthy groups for mutant allele carriers compared with those with AA genotype. The frequency of the C allele was higher among patients than the control group and statistical analysis showed that there was a significant association between the C allele and the increased risk of breast cancer (Table 2).

For TaqI polymorphism, alleles with thymine nucleotide were more abundant than other allele. The frequency distribution of TC genotype in healthy and patient groups was 39.33% and 45.33%, respectively, while this ratio for CC mutant genotype was 8.00% and 14.00%, respectively. Statistical analysis revealed that there was a significant association between CC genotype and increased risk of breast cancer (Table 2). Statistical analysis showed a significant difference between the patient and healthy groups in the carriers of the C allele compared with those with the TT genotype. The frequency of the C allele was higher among patients than the control group and statistical analysis showed that there was a significant association between the C allele and the increased risk of breast cancer (Table 2).

*Stratified analysis*

The genotypes distribution for ApaI and TaqI polymorphisms were evaluated for tumor size and lymph node metastasis (Table 3). With regard to ApaI polymorphism, we observed that there are no significant associations between this polymorphism and tumor size of breast cancer in three genetic models (AC vs. AA: OR: 0.71, 95%CI= 0.34-1.50, p= 0.373; CC vs. AA: OR= 0.55, 95%CI= 0.19-1.62, p= 0.277; AC+CC vs. AA= OR= 0.67, 95%CI= 0.33-1.36, p= 0.267). In addition, we did not find any significant association between ApaI polymorphism and lymph node metastasis of breast cancer in three mentioned genetic models. As shown in Table 4, similar results were observed for the TaqI polymorphism. Our data revealed that this polymorphism could not affect the tumor size and lymph node metastasis. Genotypes distribution showed that TC, CC, and TC+CC are not associated with the tumor size of breast cancer. The same results were observed for lymph node metastasis in three genetic models (TC vs. TT: OR= 0.985, 95%CI= 0.49-2.00, p= 0.967; CC vs. TT: OR= 0.59, 95%CI= 0.22-1.60, p= 0.300; TC+CC vs. TT: OR= 0.87, 95%CI= 0.45-1.69, p= 0.682).

**Table 1:** The Sequence of Primers and PCR Program

SNP name	Primer name	5' to 3' oligonucleotide	PCR program
ApaI	Apa-F	5'- GGACAGAGCATGGACAGGGAGC	94°C (5′), 94°C (40″), 61°C (40″), 72°C (40″), 35 cycles, 72°C (5′)
TaqI	Taq-R	5'- GGGCGTTAGCTTCATGCTGCAC	

**Table 2:** Association between VDR-ApaI Polymorphism and Breast Cancer Risk

SNP ID	Genotype/ Allele	No. and Percentage		χ2	OR (95% CI)	*P*-value
		Control (n= 150)	Case (n= 150)			
*ApaI* *(rs7975232)*	AA	72 (48.00%)	53 (35.33%)	-	-	-
	AC	60 (40.00%)	73 (48.67%)	4.02	1.65 (1.0101 to 2.7045)	0.046*
	CC	18 (12.00%)	24 (16.00%)	2.75	1.81 (0.8935 to 3.6718)	0.099
	AC+CC	78 (52.00%))	97 (64.67%)	4.95	1.69 (1.0629 to 2.6851)	0.027*
	A	204 (68.00%)	179 (59.67%)	-	-	-
	C	96 (32.00%))	121 (40.33%)	4.51	1.44 (1.0278 to 2.0076)	0.034*
*TaqI* *(rs731236)*	TT	79 (52.67%)	61 (40.67%)	-	-	-
	TC	59 (39.33%)	68 (45.33%)	2.65	1.49 (0.9209 to 2.4193)	0.104
	CC	12 (8.00%)	21 (14.00%)	4.31	2.27 (1.0348 to 4.9639)	0.041*
	TC+CC	71 (47.33%)	89 (59.33%)	4.34	1.62 (1.0279 to 2.5640)	0.038*
	T	217 (72.33%)	190 (63.33%)	-	-	-
	C	83 (27.67%)	110 (36.67%)	5.57	1.51 (1.0279 to 2.5640)	0.019*

*Significant difference between patient and control group; OR, odds ratio; CI, confidence interval

**Table 3:** The Association Analysis between VDR-ApaI Variation and Two Clinical Characteristics of Breast Cancer

Characteristics	Genotype distributions			
	AA	AC	CC	AC+CC
Tumor size (cm)				
≥2/<2	20/33	22/51	6/15	28/69
OR (95% CI)	1.0 (reference)	0.71 (0.34-1.50)	0.55 (0.19-1.62)	0.67 (0.33-1.36)
*P*-value		0.373	0.277	0.267
Lymph node metastasis				
Yes/No	29/24	42/31	17/7	59/38
OR (95% CI)	1.0 (reference)	1.12 (0.55-2.29)	2.01 (0.72-5.65)	1.28 (0.65-2.53)
*P*-value		0.753	0.185	0.468

Cm, centimeter, OR, odds ratio, CI, confidence interval

**Table 4:** The Association Analysis between VDR-TaqI Variation and Two Clinical Characteristics of Breast Cancer

Characteristics	Genotype distributions			
	TT	TC	CC	TC+CC
Tumor size (cm)				
≥2/<2	15/46	23/45	10/11	33/56
OR (95% CI)	1.0 (reference)	1.57 (0.73-3.38)	0.93 (0.36-2.40)	0.60 (0.33-1.09)
*P*-value		0.252	0.88	0.095
Lymph node metastasis				
Yes/No	37/24	41/27	11-Oct	51/38
OR (95% CI)	1.0 (reference)	0.985 (0.49-2.00)	0.59 (0.22-1.60)	0.87 (0.45-1.69)
*P*-value		0.967	0.3	0.682

Cm, centimeter, OR, odds ratio, CI, confidence interval

## Discussion

The role of genetic and environmental risk factors on the development of breast cancer susceptibility was investigated widely (Mavaddat et al., 2010; Nickels et al., 2013). In breast cancer, the genetic, environmental, behavioral, and the combined effects of risk factors are involved (Nickels et al., 2013). Research has shown that vitamin D has a protective effect against breast cancer and its deficiency may be a risk factor for the onset and progression of breast cancer (Jurutka et al., 2001). It has been revealed that vitamin D could affect cell proliferation and differentiation in cancers (Colston et al., 1981). 

In the current study, we investigated the association of ApaI and TaqI polymorphisms in the *VDR *gene with breast cancer in a case-control study and a stratified analysis. In the case of ApaI polymorphism, there was a significant association between AC genotype and increased risk of breast cancer. Our results showed a significant difference between the patient and healthy groups for C allele carriers compared to those with AA genotype. The frequency of the C allele was higher in the patient group than the control group and the statistical analysis showed that there was a significant association between C allele and increased risk of breast cancer. In addition, *TaqI* polymorphism was associated with breast cancer risk in some genetic models. But, the stratified analysis based on tumor size and lymph node metastasis revealed no significant associations for both studied SNPs. 

Similar studies have been conducted in recent years that have reported different results. For example, Ahmed et al., (2019) investigated the association of this polymorphism with the risk of breast cancer in the Ethiopian female population, whose results showed that the *ApaI* polymorphism was not associated with breast cancer in any genetic model. But, subgroup analysis showed that the CC genotype is correlated with a high concentration of 25 (OH) D3 in plasma in the tamoxifen receiving group (Ahmed et al., 2019). However, El-Shorbagy et al., (2017) reported that the ApaI polymorphism in the *VDR* gene is a genetic risk factor for breast cancer in the Egyptian female population (El-Shorbagy et al., 2017). Concerning the TaqI polymorphism, similarly, Ahmed et al., (2019) reported that this genetic variant was not associated with breast cancer risk (Ahmed et al., 2019) But, El-Shorbagy et al., (2017) reported that this polymorphism was associated with risk of breast cancer. Different results from different studies indicate that the association of *VDR* gene polymorphisms with breast cancer risk may be affected by some factors including race, diet, lifestyle, and environmental factors.

Previous studies have shown that vitamin D plays an important role against several cancers including breast cancer. The vitamin D3 receptor (VDR) as a nuclear receptor complexed with its ligand 1-α, 25-dihydroxycholecalciferol (1, 25(OH) 2 D3) could modulate gene expression. The *VDR* expressed in the mammary gland, normally. It functions against the estrogen-driven proliferation and maintains differentiation, and it may participate in negative-growth regulation of mammary epithelial cells.* VDR* gene SNPs could be studied as an important factor, because the variants of *VDR* gene may affect receptor function (Welsh et al., 2003). Therefore, it can be concluded that VDR polymorphisms may influence vitamin D metabolism and 25(OH) D levels (Tiosano et al., 2001). There are convincing data of the *VDR *gene in different studies, but to obtain sufficient information regarding the use of vitamin D supplements to prevent or treat breast cancer, is needed more research. 

Assessing the molecular effects of genetic polymorphisms on the structure and function of mRNA and protein in vitro and in vivo is time-consuming and costly (Zamani-Badi et al., 2018; Zamani-Badi et al., 2019). But computational methods using bioinformatics tools can be a useful way for molecular analysis especially to evaluate the effects of genetic polymorphisms (Karimian et al., 2015; Noureddini et al., 2018; Tameh et al., 2018; Mobasseri et al., 2019; Karimian et al., 2020). In silico analysis is a useful tool to evaluate the effects of SNPs on protein and mRNA structure (Salimi et al., 2017; Bafrani et al., 2019). Therefore, this tool would be an appropriate approach to study the effects of *TaqI *and *ApaI* on the *VDR* gene function.

In conclusion, our case-control study shows that TaqI and ApaI polymorphisms of the *VDR* gene are associated with breast cancer risk. Therefore, these genetic variants can be risk factors for breast cancer. Besides, these polymorphisms can be considered as potential biomarkers for screening women susceptible to breast cancer. However, there are several limitations to the present study that should be considered. The *VDR* gene interacts with many other genes, so gene-gene interactions, as well as gene-environment interactions, may affect the effects of studied genetic variations. In our study, the absence of baseline data such as sunlight exposure, skin color, and family history may affect the accuracy of the association of *TaqI* and *ApaI* polymorphisms in the *VDR* gene with breast cancer risk. To obtain more accurate information, further studies on a larger sample size in combination with different environmental and genetic factors are needed.
